# Development of a Clinic Screening Tool to Identify Burdensome Health-Related Issues Affecting People Living With HIV in Spain

**DOI:** 10.3389/fpsyg.2021.681058

**Published:** 2021-06-09

**Authors:** Maria José Fuster-RuizdeApodaca, Kelly Safreed-Harmon, Marta Pastor de la Cal, Ana Laguia, Denise Naniche, Jeffrey V. Lazarus

**Affiliations:** ^1^Sociedad Española Interdisciplinaria del Sida (SEISIDA), Madrid, Spain; ^2^Universidad Nacional de Educación a Distancia (UNED), Madrid, Spain; ^3^Barcelona Institute for Global Health (ISGlobal), Hospital Clínic, University of Barcelona, Barcelona, Spain; ^4^Bizkaisida, Bilbao, Spain

**Keywords:** HIV, patient-reported outcome measure (PROM), health-related quality of life, symptom assessment, health measurement instrument, psychometrics, Spain

## Abstract

**Background:**

Numerous health-related issues continue to undermine the health and health-related quality of life (HRQoL) of people living with HIV (PLHIV). We developed a clinic screening tool (CST-HIV) for the purpose of identifying these issues in routine specialist clinical care in Spain.

**Methods:**

We used the following established instrument development procedures: (1) a literature review; (2) four focus group discussions (FGDs), two that convened 16 expert HIV care providers, and two that convened 15 PLHIV; (3) prioritisation, selection and definition of constructs (health-related issues) to include in the CST-HIV and drafting of initial item pool; and (4) a pilot study to analyse psychometric properties and validity of items and to determine which to retain in the final CST-HIV. The FGD interview scripts incorporated an exercise to prioritise the health-related issues perceived to have the greatest negative effect on HRQoL. The online questionnaire used for the pilot study included the pool of CST-HIV items and validated measures of each construct.

**Results:**

We identified 68 articles that reported on factors associated with the HRQoL of PLHIV. The most burdensome health-related issues identified in the FGDs related to stigma, socioeconomic vulnerability, sleep/fatigue, pain, body changes, emotional distress, and sexuality. Based on the literature review and FGD findings, we selected and defined the following constructs to include in the initial CST-HIV: anticipated stigma, emotional distress, sexuality, social support, material deprivation, sleep/fatigue, cognitive problems, and physical symptoms. Two researchers wrote six to eight items for each construct. Next, 18 experts rated 47 items based on their clarity, relevance, and representativeness. Pilot testing was carried out with 226 PLHIV in Spain. We retained 24 items based on empirical criteria that showed adequate psychometric properties. Confirmatory factor analysis confirmed the eight-factor structure with a good fit to the data (RMSEA = 0.035, AGFI = 0.97, CFI = 0.99). We found strong positive correlations between the instrument’s eight dimensions and validated measures of the same constructs. Likewise, we found negative associations between the dimensions of the CST-HIV and HRQoL.

**Conclusion:**

The CST-HIV is a promising tool for use in routine clinical care to efficiently identify and address health-related issues undermining the HRQoL of PLHIV.

## Introduction

Widespread access to antiretroviral therapy (ART) has enabled many people living with HIV (PLHIV) to control their infection on a long-term basis. The life expectancy of PLHIV now approaches that of the general population in resource-rich settings and has greatly increased in resource-poor settings as well (Antiretroviral Therapy Cohort Collaboration, 2017; Teeraananchai et al., 2017). However, numerous issues undermine the well-being of PLHIV, including PLHIV who are stable on ART.

Multimorbidity is more prevalent among PLHIV than members of the general population, with commonly occurring comorbidities including mental health disorders and ageing-related non-communicable diseases such as cardiovascular, liver and kidney disease (Chuah et al., 2017; Maciel et al., 2018; Smit et al., 2015). PLHIV have a high burden of symptoms of ill health such as pain, fatigue and gastrointestinal problems (Harding et al., 2010; Wilson et al., 2016; Ibarra-Barrueta et al., 2019). They furthermore face an array of challenges to their psychosocial and material well-being (Bristowe et al., 2019; Public Health England, 2020). HIV-related stigma and discrimination have far-reaching ramifications in terms of mental health, medication adherence, health-seeking behaviour, social relationships, employment and other areas of people’s lives (Sweeney and Vanable, 2016; Wagener et al., 2017; Ikeda et al., 2019). PLHIV also must grapple with the emotional and practical demands of living with a complex chronic health condition that requires lifelong ongoing treatment.

In this context, it is notable that a large study in the United Kingdom found poorer health-related quality of life (HRQoL) outcomes among PLHIV than among the general population (Miners et al., 2014). This difference persisted even for the subgroup of PLHIV who were virally suppressed. Other research has found poor HRQoL outcomes in PLHIV populations to be associated with a wide range of factors, including pain, insomnia, mental health disorders and HIV-related stigma (Degroote et al., 2014; Sabin et al., 2018; Andersson et al., 2020; Kunisaki et al., 2021).

People living with HIV who have responded well to ART typically are advised to see their healthcare providers for clinical monitoring two to four times per year. These routine clinic visits present an important window of opportunity for healthcare providers to identify and address some of the problems that can contribute to poor HRQoL. However, PLHIV often encounter communication barriers with their healthcare providers and may not feel that providers are responsive to their healthcare priorities (Antunes et al., 2020; Fredericksen et al., 2020a; Okoli et al., 2020). Furthermore, providers may overlook important symptoms (Edelman et al., 2011).

In recent years, the World Health Organization (WHO) and many health systems increasingly have promoted person-centred care, which WHO describes as being “organised around the comprehensive needs of people rather than individual diseases” (McCormack et al., 2015; World Health Organization (WHO), 2016). One means of promoting good communication about people’s healthcare needs is to ask patients to complete surveys known as patient-reported outcome measures (PROMs) (Wheat et al., 2018; Fredericksen et al., 2020b). There are validated PROMs focusing on numerous aspects of health and well-being, including generic PROMs designed for all patient populations as well as PROMS that reflect the concerns of patients with specific diseases and conditions including HIV.

A 2017 review of HIV-specific PROMs identified 117 validated instruments for measuring patients’ perceptions of their health and related issues in areas such as medication adherence, symptoms, psychological challenges, HIV-related stigma, social support, and sexual and reproductive health (Engler et al., 2017). Because these instruments typically focus on narrow topics, it would be necessary to use multiple instruments to learn about different aspects of a patient’s well-being. The time-intensive nature of such an approach points to a need for broadly focused PROMs that are short enough to be easily integrated into routine clinic visits, enabling healthcare providers to quickly determine which of many potential health-related problems should be addressed in these visits. Despite the contribution that this type of PROM could make to routine clinical care, this remains an area under development. The only such instrument that we are aware of in the HIV field is currently being developed by Bristowe et al. (2019, 2020), with the content of the instrument guided by qualitative research involving PLHIV and other key stakeholders in England and Ireland.

The present study is part of a broader research project to improve the HRQoL and the long-term health of PLHIV in Spain and Italy. It constitutes the first stage of the research, and its aim is to develop a brief Spanish clinic screening tool (CST-HIV) that can be used in routine clinical care to identify problems that undermine the HRQoL of PLHIV. This paper reports the process of developing the instrument to ensure its content and face validity, describes the psychometric properties of the instrument, and presents the evidence of construct and criterion validity that we obtained when we piloted the instrument.

## Materials and Methods

### Study Design

This study comprised several steps, including a literature review, a qualitative study, an item design process, a cognitive debriefing study, and a pilot cross-sectional *ex post*-*facto* study to analyse the psychometric properties of the initial version of the CST-HIV. Table 1 summarises the research design, procedures and participants involved. All of these steps will be detailed in the following sections.

**TABLE 1:** Summary of research design.

Steps		Procedures	Participants involved
Step 1	Literature review	Identification of initial domains	Authors
Step 2	Qualitative study with focus groups	Identification of initial domains	*N* = 15 PLHIV *N* = 16 experts *N* = 31 Total
Step 3	Development of initial pool of items	Definition of constructs and drafting of items	*N* = 3
		Expert assessment and inter-rater process	*N* = 18
		Cognitive debriefing	*N* = 8
Step 4	Pilot study	Assessment of psychometric properties and validity of items	*N* = 226

*PLHIV, people living with HIV.*

### Participants

A total of 31 persons participated in the qualitative study to identify the initial dimensions of the CST-HIV. Sixteen of them were expert service providers from diverse disciplines (physicians, nurses, psychiatrists, psychologists, and staff of non-governmental organisations [NGOs]). The remaining 15 were PLHIV. Six of the experts and also six of the PLHIV were cis-women. Among PLHIV, one transgender woman also participated. The other participants were cis-men.

A total of 18 multidisciplinary experts from diverse disciplines and areas of expertise, three of whom were PLHIV, participated in the expert assessment and inter-rater process to develop the initial pool of items.

Eight PLHIV, five men and three women, participated in the cognitive debriefing of the CST-HIV items. Next, we conducted the pilot study investigating the item pool’s psychometric properties in a sample of 226 PLHIV from different regions of Spain. The sample size was determined in accordance with the sample size requirements for carrying out confirmatory factor analysis (Bentler and Chou, 1987). Since these requirements call for 10 participants per item, and we anticipated that the final number of CST-HIV items would be between 21 and 24, our target sample size was between 210 and 240 PLHIV. The inclusion criteria were having an HIV-positive diagnosis, being at least 18 years old, and not having any severe psychiatric or cognitive disorders. Excluding people with such disorders is standard in studies in which participants complete self-administered surveys since the presence of such disorders could affect one’s cognitive capacity to understand questions and provide reliable responses.

Table 2 shows the sociodemographic and clinical characteristics of pilot study participants. Most of them were male and homosexual, and the most commonly reported mode of HIV infection was sexual intercourse. The mean age was 44. Approximately one-third of the participants had a university degree, and 39% were employed. Sixty-eight percent reported having a personal monthly income of €900 or less. The immunological and virological HIV status of most participants were good.

**TABLE 2:** Characteristics of pilot study participants (*N* = 226).

Sociodemographic and clinical variables	% (*n*)
Age, mean (*M* ± *SD*)	43.81 ± 11.15
**Gender**	
Male	75.7 (171)
Female	21.7 (49)
Transgender	1.3 (3)
Other	1.3 (3)
**Sexual orientation**	
Heterosexual	41.2 (93)
Homosexual	54.9 (124)
Bisexual	2.2 (5)
Other	1.3 (3)
No answer	0.4 (1)
**Education level**	
No education	2.2 (5)
Elementary school	19.9 (45)
High school	44.2 (100)
University degree	32.3 (73)
Other	1.3 (3)
**Work situation**	
Working	38.9 (88)
Unemployed	28.3 (64)
Retired/on disability	21.2 (48)
Other	11.5 (26)
**Personal monthly income**	
None	12.8 (29)
≤ 300 €	13.7 (31)
301–600 €	17.3 (39)
601–900 €	24.3 (55)
901–1200 €	13.7 (31)
1201–1800 €	11.9 (27)
1801–2400 €	3.1 (7)
2401–3000 €	0.4 (1)
3001–4500 €	0.9 (2)
No answer	1.8 (4)
**Housing**	
Own home (rent or own)	56.6 (128)
Family home	12.8 (29)
Shared home	16.8 (38)
Someone else’s home	1.3 (3)
Shelter/institution	6.6 (15)
Other	5.8 (13)
**HIV transmission route**	
Sexual intercourse	78.3 (177)
Sharing injection materials	10.6 (24)
Unknown	8.8 (20)
Other	2.2 (5)
**CD4 cell count, cells/mm^3^**	
≤ 200	7.5 (17)
201–400	7.1 (16)
> 400	53.5 (121)
Unknown	31.9 (72)
Duration of infection, years, mean (*M* ± *SD*)	14.18 ± 10.47
Undetectable plasma viral load	92.5 (209)

*Data in percentages unless otherwise stated.*

### Procedure

This research took place from April 2019 to October 2020. The Ethics Committee of the Hospital Clínic of Barcelona, Spain, approved all research procedures. Participants in all phases signed informed consent forms before data collection began.

The HIV Clinic Screening Tool (CST-HIV) was developed through the following well-established methodological steps (Eignor, 2001; Revicki et al., 2007).

Firstly, we conducted an exploratory literature review to obtain information about issues that undermine the well-being of PLHIV and to identify themes that would warrant further exploration in focus group discussions (FGDs). We identified English-language peer-reviewed articles and conference abstracts indexed in PubMed using search strings that addressed two major lines of research: HIV symptom burden and predictors of HRQoL in PLHIV. We used appropriate selection criteria to identify the studies of greatest relevance to our study (e.g., studies reporting on adult PLHIV who are taking ART and studies reporting on the symptom burden in PLHIV from 2010 onward, in recognition that the symptom profile has changed in accordance with ART improvements). We used Scopus and ResearchGate to identify articles that cited a key source about the widely used HIV Symptom Index (Justice et al., 2001). Selected references were compiled in tables to identify evidence regarding burdensome symptoms and predictors of HRQoL in PLHIV.

Drawing on literature review findings, we conducted a qualitative study using the FGD methodology to obtain the perspectives of PLHIV and other key informants regarding the most burdensome health-related problems facing PLHIV. We carried out four FGDs. Two of them enrolled HIV service providers (*n* = 8 per FGD), and the other two enrolled PLHIV (*n* = 8 and *n* = 7). Participants in the service provider FGDs were selected via purposive sampling to ensure the representation of different types of providers such as physicians, nurses, psychiatrists, psychologists, and NGO staff. Service providers worked in Barcelona, Bilbao, Madrid, Seville, and Valencia. Participants in the PLHIV FGDs were selected via purposive sampling to ensure diverse epidemiological profiles in terms of age, sex, sexual orientation, and drug use history. One PLHIV FGD was comprised of clients of an NGO providing HIV services in Barcelona, and the other PLHIV FGD was comprised of patients at the HIV outpatient clinic of a large Barcelona university hospital. FGDs took place in April and May 2019, with each one lasting approximately two hours. Facilitators used semi-structured scripts with open-ended questions and prompts to guide the discussions.

The next step in the development of the CST-HIV consisted of developing a pool of potential items. Based on findings from the FGDs and the literature reviews, three members of the research team selected the most prevalent and burdensome health-related problems undermining the HRQoL of PLHIV. Also, they defined the constructs (the health-related problems) after deliberation (Nunnally and Bernstein, 1994). Items were developed to measure each construct, following psychometric recommendations (Osterlind, 1989; Haladyna et al., 2002), and the response format for the items was decided. A team of 18 multidisciplinary experts rated the items based on their clarity, relevance and representativeness. Based on the experts’ ratings and comments, items were selected and reworded as appropriate to create the initial item pool. A cognitive debriefing study was then carried out, in which eight PLHIV rated the understandability of the items. These participants were members of the NGO collaborators in the research.

Finally, we conducted a pilot study to assess the initial items’ psychometric properties and to select those that would be part of the final CST-HIV. We recruited participants through NGO collaborators, and we asked those who agreed to participate to complete an online questionnaire using Qualtrics^1^, a private online survey development platform.

### Measures

For the qualitative study, we designed a semi-structured FGD script addressing two central questions: (1) “In your opinion, what are the health-related problems that have the most significant negative effect on the quality of life of PLHIV?”; and (2) “Among the problems that you have identified, what do you think are the most important ones to include in a short diagnostic questionnaire?” All FGD participants were also asked to carry out a prioritisation exercise in which they selected what they believed to be the most burdensome issues from among all issues identified during the discussions.

The online questionnaire used for the pilot study included the 40 items selected after the inter-rater process. We selected brief instruments to measure preliminary evidence of the convergent validity of each CST-HIV dimension. We chose instruments according to their psychometric properties, validity, and availability of cut-off points. When a Spanish version of an instrument was available, we used it. When it was not, we conducted a backward translation of the instrument. The questionnaire included the following instruments:

#### Anticipated Stigma

The factors of disclosure concerns and public stigma of the Spanish Stigma Scale measured through 13 items were used (Fuster-RuizdeApodaca et al., 2015). Results of the Spanish adaptation of the instrument indicated that these two factors could be grouped in a latent second-order dimension related to internalised stigma (Fuster-RuizdeApodaca et al., 2015). The scale is rated on a four-point response format (*1* = *strongly disagree, 4* = *strongly agree*), with higher scores indicating greater concerns.

#### Emotional Distress

We used the Patient Health Questionnaire-4 (PHQ-4) (Kroenke et al., 2009) and the Spanish version of the Hospital Anxiety and Depression Scale (HADS) (Tejero et al., 1986). The PHQ-4 is a validated ultra-brief screening tool that has a two-factor structure, one containing two anxiety items (GAD-2) and the other containing two depression items (PHQ-2). Responses are scored from 0 (*not at all*) to 3 (*nearly every day*). The total score on this measure ranges from 0 to 12. The HADS is a 14-item, self-reporting screening scale that contains two seven-item Likert scales, one for anxiety and one for depression. Each item is answered by the patient on a four-point (0–3) response category, and thus the possible scores range from 0 to 21 for anxiety and 0 to 21 for depression.

#### Sexuality

We used the PROMIS V2.0 Satisfaction with Sex Life scale (Weinfurt et al., 2015), which is part of the modular and customisable PROMIS Sexual Function and Satisfaction 2.0 measures that assess multiple components of sexual functioning. The Satisfaction with Sex Life module assesses how satisfying and pleasurable the person regards his or her sexual activities, with no constraints on how the person defines “sex life”. Items are gender-non-specific. Higher scores indicate more satisfying sexual experiences.

#### Social Support

The Duke-UNC Functional Social Support Questionnaire was selected (Broadhead et al., 1988). It is an 11-item scale measuring two dimensions of social support: confidant support and affective support. Items have a five-point Likert format response. Higher scores indicate higher social support.

#### Material Deprivation

We used the Social Exclusion Index for Health Surveys (SEI-HS) (Van Bergen et al., 2017). This instrument contains 17 items that measure four dimensions: (1) social participation; (2) normative integration; (3) material deprivation; and (4) access to basic social rights.

#### Sleep Problems

We used the Spanish version of the Insomnia Severity Index (ISI) (Bastien et al., 2001; Fernandez-Mendoza et al., 2012). This seven-item index is a reliable measure for evaluating perceived sleep difficulties. Each item is rated on a 5-point Likert scale (*0* = *no problem, 4* = *very severe problem*), yielding a total score ranging from 0 to 28.

#### Fatigue

We used the seven-item version of the Fatigue Severity Scale (FSS), which has demonstrated good psychometric properties in PLHIV (Lerdal et al., 2011). Each item is rated on a seven-point Likert-scale (*1* = *strongly disagree, 7* = *strongly agree*). The mean score is used to estimate fatigue severity.

#### Cognitive Problems

The Neuro-QoL V2.0 Cognitive Function measure was used for cognitive assessment (Lai et al., 2014). This eight-item scale measures both cognitive function concerns and abilities.

#### HRQoL

We used the HIV-specific WHOQoL-HIV-BREF measure that has been validated in Spanish (Fuster-RuizdeApodaca et al., 2019). The instrument has 31 items covering six domains: physical health; psychological health; level of independence; environmental health; social relationships; and spirituality, religion and personal beliefs (SRPB). It additionally has a general health dimension assessing one’s overall perception of one’s health and HRQoL. All items use a five-point scale. Negative items are reverse-coded for scoring. Thus, higher scores for all items indicate better HRQoL.

We also used the generic HRQoL measure EQ-5D-5L, which has five dimensions: mobility, self-care, usual activities, pain/discomfort, and anxiety/depression. Responses are provided on a five-point scale ranging from “no problems” to “extreme problems” (Herdman et al., 2011).

The online questionnaire also included a section that requested health and sociodemographic data.

### Data Analysis

To analyse the qualitative data, we performed directed content analysis (Mayring, 2000) using MAXQDA 12 software. The FGDs were transcribed, reviewed for accuracy, and coded. Inductive and deductive coding were used to identify relevant concepts, and an analysis of these concepts led to the identification of key categories and subcategories of health-related problems. We also performed a quantitative analysis of the qualitative data to determine the number of times each code and category was used. Two analysts discussed and agreed on the data categorisation, with inconsistencies resolved by consensus. Following the coding of the FGD content, all research team members reviewed and approved the final categorisation of data.

To analyse the content validity of the initial pool of items evaluated in the inter-rater process, we calculated the Osterlind Index (Osterlind, 1989) for the items’ representativeness and relevance scores. Representativeness and relevance items had a three-point ordinal response (high, medium, low). There is no clear criterion regarding a cut-off point for this index; some use 0.5 and others 0.75 depending on the objective. We used a strict criterion in most dimensions, selecting items with an Osterlind Index of up to 0.75.

In the pilot study, we assessed the psychometric properties of the initial CST-HIV item pool based on empirical criteria. We assessed the floor and ceiling effects, the internal consistency, the reliability, and the validity index of each dimension (Kline, 2013). Most items in the online questionnaire in the Qualtrics survey platform were programmed for compulsory completion. Thus, there were no missing values in the variables collected.

Next, to test the construct validity, first-order confirmatory factor analysis (CFA) was used to assess the retained CST-HIV items’ fit with the theoretical proposed structure. Due to the ordinal nature of our data and the sample size, we chose the robust unweighted least-squares extraction method (ULS) (Batista-Foguet and Coenders, 2000; Holgado-Tello et al., 2009; Holgado–Tello et al., 2010). Although the weighted least squares method also could be used, we did not use it because of the instability of its inverse matrix when the models have more than ten variables or a moderate sample size (Holgado-Tello et al., 2018; Holgado-Tello et al., 2009; Satorra, 1990). The goodness of fit was evaluated using several absolute and relative fit indices, including the goodness of fit index (GFI), the adjusted goodness of fit index (AGFI), the comparative fit index (CFI), the standardised root mean square residual (SRMR) and the standardised root mean square error of approximation (RMSEA). A model is considered to have a good fit when the goodness of fit indices (GFI and AGFI) and CFI are greater than 0.90, RMSEA is lower than 0.08, and SRMR is lower than 0.08 (Hu and Bentler, 1995).

We then calculated reliability and construct statistics of the CST-HIV including the Cronbach’s alpha coefficient to assess internal consistency, the average extracted variance (AVE) to assess convergent validity, and the Jöreskog rho (Omega) to assess construct reliability (Fornell and Larcker, 2016). Cronbach’s alpha coefficients between 0.70 and 0.90 are adequate, and between 0.60 and 0.70 are acceptable (Kline, 2013). AVE values greater than 0.50 indicate convergent validity, and Omega coefficients between 0.70 and 0.90 are considered to represent acceptable construct reliability (Campo-Arias and Oviedo, 2008), although in some circumstances, values higher than 0.65 can be accepted (Katz, 2006).

Convergent and concurrent validity were analysed by calculating the Pearson correlation between each CST-HIV dimension and the validated instruments used to measure the constructs and HRQoL. We expected each dimension to correlate positively with its convergent criterion measure and negatively with HRQoL.

Regarding the data analysis software, LISREL (LInear Structural RELations) 8.7 and its companion preprocessor programme PRELIS for Windows were used for the CFAs (Jöreskog and Sörbom, 1996). IBM SPSS Statistics 22 (IBM Corp, 2013) was used for the remaining analyses.

## Results

### Step One – Identification of Dimensions to Include in the CST-HIV: Literature Review

The literature review on the HIV symptom burden identified five articles and two conference abstracts that were relevant to the current study. The symptoms that were most commonly reported to be highly prevalent in PLHIV were sleep-related problems, fatigue, and muscle/joint pain (Erdbeer et al., 2014; McGowan et al., 2014; Wilson et al., 2016; Schnall et al., 2018; Cioe et al., 2019; Ibarra-Barrueta et al., 2019; Schnall et al., 2019). Other highly prevalent symptoms observed in some studies included anxiety, depression, sexual dysfunction, changes in body appearance, and gastrointestinal problems (Erdbeer et al., 2014; Wilson et al., 2016; Schnall et al., 2018; Ibarra-Barrueta et al., 2019).

The HRQoL literature review identified a large body of relevant research on factors associated with HRQoL outcomes in PLHIV, including a 2014 review article (Degroote et al., 2014). We analysed the findings of the review article and 68 additional articles that reported on more recent studies. We observed that one of the factors most commonly reported to be associated with positive HRQoL outcomes in PLHIV is social support (Bekele et al., 2013; Emlet et al., 2013; Slater et al., 2013; Dalmida et al., 2015; George et al., 2016; Nideröst and Imhof, 2016; den Daas et al., 2019). Two factors associated with negative HRQoL outcomes in many studies are depression and material insecurity (e.g., unemployment, financial problems, unmet needs for food and housing) (Douab et al., 2014; Dalmida et al., 2015; Ballester-Arnal et al., 2016; George et al., 2016; Nideröst and Imhof, 2016; Catalan et al., 2017; Logie et al., 2018; Sok et al., 2018; Olson et al., 2019). Other factors associated with negative HRQoL outcomes in some studies included comorbidity, stigma and HIV disclosure concerns (Emlet et al., 2013; Slater et al., 2013; Fekete et al., 2016; George et al., 2016; Nideröst and Imhof, 2016; Logie et al., 2018; Reinius et al., 2018). A high symptom burden was also associated with negative HRQoL outcomes, as were specific symptoms such as body disfigurement, memory difficulties and sexual functioning (Ballester-Arnal et al., 2016; George et al., 2016; Brandt et al., 2017; den Daas et al., 2019; Olson et al., 2019).

### Step Two – A Qualitative Study With Focus Groups to Identify the Most Burdensome Health-Related Problems Undermining HRQoL in PLHIV

Focus group discussion participants identified many issues that impact the HRQoL of PLHIV. The issue raised most frequently by both PLHIV and healthcare providers was stigma/discrimination (*n* = 150 segments coded), with people commenting far more on this issue than on physical symptoms or emotional problems. The category of physical symptoms was the second-most frequently discussed (*n* = 83 segments coded). The physical symptom noted most often was sleep problems. Other physical symptoms that were frequently mentioned included fatigue, pain, body fat changes, and neurocognitive problems. Both PLHIV and healthcare providers emphasised the importance of psychological well-being (*n* = 67 segments coded). They often commented on emotional distress in general terms rather than naming specific disorders, although depression and anxiety were mentioned numerous times. Healthcare providers, and to a lesser extent PLHIV, called attention to sexuality-related problems such as lack of libido, sexually transmitted infections and general sexual dissatisfaction. When PLHIV addressed sexuality-related problems, they often linked these problems to their perceptions about HIV-related stigma.

### Step Three – Development of Potential CST-HIV Items

The initial item pool was developed through the following steps:

(a) Selection and definition of the constructs to include in the CST-HIV. A theoretical conceptualisation of the selected health-related problems undermining HRQoL was carried out, taking into account the content analysis of the FGDs and the literature review. A total of eight constructs were selected: anticipated stigma, emotional distress, sexuality, social support, material deprivation, sleep/fatigue, cognitive problems, and physical symptoms. Three members of the research team wrote independent definitions for the constructs. They then met to reach agreement about definitions and about the essential components that should be included in the instrument.
(b) Development and writing of items. First, we conducted a review of validated instruments measuring the constructs selected for inclusion in the CST-HIV. The same three researchers selected the items that most closely represented the components of each construct. Drawing on these items and the definitions of constructs, two Spanish researchers adapted or wrote six to eight items for each construct. Psychometric recommendations for the development of items were followed (Nunnally and Bernstein, 1994), with the following criteria taken into account: clarity (i.e., items should be written in short, simple and intelligible sentences, and should avoid excessive generality); relevance (i.e., content should be clearly related to the construct); and representativeness (i.e., items should be representative of the construct). This process yielded an initial pool of 47 items.
(c) Expert assessment and inter-rater process. The 18 participating experts rated the items based on their clarity, relevance and representativeness. They also assessed whether the items required modification, and provided further input in comments. This process led to the elimination of seven items. Sixteen other items were modified in response to suggestions from experts. The item pool to be evaluated in the pilot study was comprised of 40 items. Table 3 shows the items and their Osterlind Index scores for representativeness/relevance. All of the experts agreed on the five-point response format that was proposed for the items.

**TABLE 3:** Psychometric properties of the initial pool of items of the CST-HIV.

CST-HIV dimensions and items	Osterlind Index (representativeness/relevance)	Mean ± SD	Skewness	Kurtosis	Corrected item-domain correlation	Cronbach’s α if item is deleted	Reliability Index	Validity Index
**Anticipated Stigma^*a*^ (α = 0.877)**								
**During the past month, to what extent have you been worried…**								
S1… about telling someone you have HIV?^1^	0.94/1.00	2.62 ± 1.51	0.40	–1.30	0.694	0.859	1.04	0.84
S3… about people judging you if they learn you have HIV?^1^	1.00/0.94	2.95 ± 1.45	0.06	–1.39	0.824	0.807	1.19	0.98
S4… about the idea that you can’t find a partner because you have HIV?	0.94/0.94	2.66 ± 1.43	0.32	–1.24	0.618	0.886	0.89	0.74
S5m… about people rejecting you for having HIV?^1,2^	1.00/1.00	2.86 ± 1.43	0.12	–1.33	0.815	0.812	1.16	0.97
**Emotional distress^*b*^ (α = 0.901)**								
**During the past month, how often…**								
E1m… have you had negative feelings? (for example, sadness, despair, low spirits, or anxiety?^2^)	1.00/1.00	3.04 ± 1.01	–0.35	–0.76	0.789	0.872	0.79	0.64
E2m… have you felt anxiety?^1^	0.88/0.89	2.94 ± 1.11	–0.33	–0.82	0.800	0.869	0.88	0.75
E3m… have you felt sadness or discouraged?^1,2^	0.88/0.83	2.95 ± 1.06	–0.33	–0.85	0.825	0.866	0.86	0.67
E5… have you felt fearful of the future?^1^	0.76/0.83	3.01 ± 1.24	–0.09	–0.99	0.744	0.881	0.92	0.69
E6m… have you been concerned for your future because of having HIV?^2^	0.81/0.88	2.74 ± 1.33	0.14	–1.19	0.647	0.907	0.86	0.66
**Sexuality^*a*^ (α = 0.734)**								
**During the past month…**								
Sx1m… how satisfied have you felt with your sex life?^1,2,3^	0.82/0.94	3.03 ± 1.21	–0.23	–0.96	0.310	0.736	0.37	0.88
Sx2… has your sex drive or interest in sex decreased?^1^	1.00/1.00	2.72 ± 1.26	0.02	–1.10	0.380	0.720	0.48	0.52
Sx3m… how difficult has it been to start an intimate or sexual relationship with a new partner?^2^	0.88/0.94	2.83 ± 1.47	0.10	–1.37	0.510	0.685	0.75	0.48
Sx4m… how fearful have you been of being rejected by a sexual partner for having HIV?^2^	0.94/0.94	2.96 ± 1.52	0.04	–1.46	0.616	0.649	0.94	0.29
Sx5… how worried have you been about transmitting HIV to a sexual partner?	1.00/1.00	2.84 ± 1.66	0.14	–1.64	0.458	0.700	0.76	0.06
Sx6… has HIV negatively affected your sex life?^1^	0.94/0.94	2.52 ± 1.33	0.39	–1.03	0.572	0.670	0.76	0.50
**Social Support^*b*^ (α = 0.837)**								
**During the past month, how often…**								
SS1m… have you had people around you whom you can lean on in case of need?^1,2,3^	0.88/0.82	3.62 ± 1.18	–0.39	–0.85	0.699	0.787	0.82	0.61
SS2… have you had someone you trust to speak to about your problems?^1,3^	1.00/1.00	3.61 ± 1.21	–0.45	–0.75	0.657	0.800	0.79	0.58
SS3… have people made you feel loved?^1,2^	0.82/0.89	3.83 ± 1.08	–0.67	–0.27	0.739	0.777	0.80	0.63
SS4… have you felt isolated from other people?	1.00/1.00	2.49 ± 1.01	0.13	–0.81	0.486	0.843	0.49	0.48
SS6… have you felt alone?	0.82/0.83	2.83 ± 1.15	–0.10	–0.89	0.622	0.809	0.71	0.55
**Material deprivation^*a*^ (α = 0.774)**								
**During the past month…**								
Ex1… how concerned have you been about your economic situation?	0.94/0.89	3.45 ± 1.31	–0.44	0.96	0.580	0.720	0.76	0.46
Ex3… have you had enough money to meet your needs?^1^	0.94/0.94	3.01 ± 1.01	–0.03	–0.80	0.646	0.703	0.65	0.58
Ex4m… how satisfied have you been with the quality of the place where you live?^1,2,3^	0.47/0.61	3.52 ± 1.10	–0.49	–0.48	0.344	0.792	0.38	0.60
Ex5m… have you had money for leisure activities?^1,2,3^	0.47/0.72	2.71 ± 1.21	0.15	–0.97	0.717	0.672	0.87	0.75
Ex6m… how worried have you been about keeping your home in the short term?^2^	0.88/0.88	2.81 ± 1.39	0.12	–1.26	0.483	0.759	0.66	0.44
**Sleep and fatigue^*a*^ (α = 0.827)**								
**During the past month…**								
SF1… have you had sleep problems?^1^	1.00/1.00	3.12 ± 1.31	–0.20	–1.04	0.571	0.815	0.75	0.89
SF3… have you had enough energy to do the things you would like to?^3^	1.00/0.94	3.22 ± 1.06	–0.16	–0.73	0.586	0.803	0.62	0.40
SF5m… have you had enough energy for your daily life activities?^2,3^	0.94/0.94	3.29 ± 0.97	–0.08	–0.62	0.627	0.794	0.61	0.44
SF7… how satisfied have you felt with the quality of your sleep?^1,3^	0.94/0.94	2.89 ± 1.14	0.01	–0.82	0.698	0.770	0.79	0.65
SF8… how tired have you felt?^1^	0.88/0.94	3.30 ± 1.01	–0.17	–0.42	0.663	0.782	0.67	0.54
**Cognitive problems^*a*^ (α = 0.924)**								
**During the past month…**								
CG1… do you feel you’ve lost memory or capacity to focus or to organise yourself?	0.94/1.00	2.80 ± 1.22	0.09	–0.96	0.769	0.911	0.93	0.71
CG2m… how difficult has it been for you to remember things?^2^	1.00/1.00	2.77 ± 1.13	0.09	–0.73	0.822	0.905	0.93	0.71
CG3… how difficult has it been for you to make decisions?	0.88/0.89	2.71 ± 1.14	0.03	–0.96	0.737	0.916	0.84	0.71
CG4… have you had difficulty thinking clearly?^1^	0.88/0.89	2.55 ± 1.15	0.23	–0.94	0.795	0.908	0.91	0.76
CG7… have you had difficulty paying attention?^1^	0.65/0.67	2.65 ± 1.17	0.12	–0.96	0.836	0.902	0.98	0.76
CG8 … do you think that it has been harder for you to learn new things?^1^	1.00/1.00	2.62 ± 1.22	0.24	–1.05	0.729	0.917	0.89	0.79
**Physical symptoms^*a*^ (α = 0.729)**								
**During the past month…**								
PS1m… have you experienced unpleasant body changes such as fat accumulation, weight gain, or weight loss?^1,2^	0.94/0.89	2.68 ± 1.26	0.19	–1.01	0.550	0.649	0.69	0.49
PS2… how worried have you been about experiencing future body changes?	0.76/0.78	3.11 ± 1.27	–0.22	–1.04	0.586	0.627	0.74	0.49
PS3… have you felt pain somewhere in your body? (for example, headache, joint pain, muscle cramps)^1^	0.89/1.00	3.04 ± 1.20	–0.24	–0.84	0.504	0.677	0.60	0.66
PS5… have you suffered digestive problems? (stomach pain, flatulence, diarrhea, nausea, or vomiting)^1^	0.94/0.89	2.62 ± 1.31	0.26	–1.13	0.440	0.715	0.58	0.58

*^1^ Item selected for final CST-HIV. ^2^ Item slightly reworded after inter-judgement process. ^3^ Reverse item. ^*a*^Response category labels: 1, None; 2, Slightly; 3, Somewhat; 4, Quite; 5, Extremely. ^*b*^Response category labels: 1, Never; 2, Rarely; 3, Sometimes; 4, Frequently; 5, Always.*

(d) Cognitive debriefing interview. Eight PLHIV completed a questionnaire containing the selected items, then reported to a member of the research team about possible difficulties in understanding the questionnaire. The items were generally regarded as relevant, accessible, and easy to understand and answer.

### Step Four – A Pilot Study to Analyse the Psychometric Properties of the CST-HIV Items

The pilot study enrolled 226 PLHIV. Data collection was carried out with the collaboration of NGOs from the following Spanish cities: Alicante, Barcelona, Bilbao, Madrid, Malaga, and Seville.

#### Assessment and Selection of the Items

Because our goal was to create a brief instrument that was feasible to use in clinical practice, we had previously decided that no more than three items should be selected for each construct. Any item was eliminated because of ceiling or floor effects. We considered each item’s reliability and validity indices to select the three items that would maximise the reliability and representation of each construct. Table 3 presents all piloted items, indicating their psychometric properties and the retained items. The Spanish wording of items is provided in Supplementary Table 1.

#### Construct Validity: Confirmatory Factor Analysis Results

The Confirmatory Factor Analysis (CFA) results confirmed the eight-factor structure with a good fit to the data. All of the standardised loadings were higher than 0.5, the level considered adequate (Green, 1978). The results of the fully standardised solution including fit indices of the model are displayed in Table 4. Table 5 reports the covariance among factors. The highest covariance was found between the physical symptoms dimension and three other dimensions: emotional distress, sleep/fatigue, and cognitive problems.

**TABLE 4:** Standardised estimations for the first-order confirmatory factor analysis model.

CST-HIV dimensions and items	Lambda (λ)
**Anticipated stigma**	
S1	0.70
S3	0.97
S5m	0.98
**Emotional distress**	
E2m	0.90
E3m	0.93
E5	0.79
**Sexuality**	
Sx1m^*a*^	0.57
Sx2	0.76
Sx6	0.82
**Social support**	
SS1m^*a*^	0.83
SS2^*a*^	0.85
SS3	0.93
**Material deprivation**	
Ex3^*a*^	0.71
Ex4m^*a*^	0.61
Ex5m^*a*^	0.97
**Sleep and fatigue**	
SF1	0.75
SF7^*a*^	0.72
SF8	0.88
**Cognitive problems**	
CG4	0.94
CG7	0.89
CG8	0.79
**Physical symptoms**	
PS1m	0.60
PS3	0.69
PS5	0.62
*SB-*χ*2*	285.09
*Degrees of freedom*	224
*p*	0.0036
*RMSEA* [90% CI]	0.035 (0.021;0.046)
*SRMR*	0.053
*GFI*	0.98
*AGFI*	0.97
*CFI*	0.99
*NFI*	0.96

*N = 226. Estimation of the robust unweighted least squares. SB-χ^2^, Satorra-Bentler chi-square; *RMSEA*, root mean square error of approximation; CI, confidence interval; *SRMR*, standardised root mean square residual; *GFI*, goodness of fit index; *AGFI*, adjusted goodness of fit index; *CFI*, comparative fit index; *NFI*, normed fit index. ^*a*^ Reversed items recoded. All factor loadings *p* < 0.05.*

**TABLE 5:** Descriptive statistics and covariances (ϕ) between the CST-HIV dimensions.

			Covariances (ϕ)							
CST-HIV dimensions	M	SD	STG	EMD	SEX	SS	MAD	SF	CG	PHYS
Anticipated stigma	8.43	4.00	1							
Emotional distress	8.90	3.03	0.48	1						
Sexuality	8.21	3.00	0.32	0.55	1					
Social support	6.95	3.10	0.34	0.29	0.39	1				
Material deprivation	8.76	2.80	0.26	0.38	0.40	0.52	1			
Sleep and fatigue	9.52	2.97	0.25	0.67	0.37	0.22	0.36	1		
Cognitive problems	7.82	3.17	0.19	0.63	0.30	0.23	0.26	0.59	1	
Physical symptoms	8.34	2.87	0.23	0.71	0.29	0.22	0.36	0.83	0.78	1

*Overall scores for each dimension, comprised by three items, ranged from 3 to 15. M, mean; SD, standard deviation; STG, anticipated stigma; EMD, emotional distress; SEX, sexuality; SS, social support; MAD, material deprivation; SF, sleep and fatigue; CG, cognitive problems; PHYS, physical symptoms.*

#### Internal Consistency

Despite the low number of items, most of the dimensions presented an alpha index of close to or ≥ 0.70, with the notable exception of the physical symptoms dimension (Table 6). However, since the number of items is crucial for Cronbach’s alpha, values lower than 0.70 for scales with only two or three items may not be considered an indicator of low consistency. As can be seen in Table 6, estimates of reliability were higher using the Jöreskog rho (omega) coefficient because the Cronbach’s alpha underestimates reliability in ordinal data (Bentler, 2009). Omega is based on the loadings rather than the correlations between the observed variables.

**TABLE 6:** Construct and reliability statistics of the CST-HIV dimensions.

CST-HIV dimension	Cronbach’s alpha	Average variance extracted	Jöreskog rho (omega)
Anticipated stigma	0.800	0.797	0.920
Emotional distress	0.866	0.766	0.907
Sexuality	0.698	0.525	0.764
Social support	0.869	0.759	0.904
Material deprivation	0.765	0.606	0.816
Sleep and fatigue	0.800	0.618	0.828
Cognitive problems	0.874	0.767	0.907
Physical symptoms	0.627	0.407	0.672

Regarding validity, we calculated the Average Variance Extracted (AVE) values for all variables. All of them except for physical symptoms were above the critical threshold of 0.50, indicating good convergent validity. The AVE measures the amount of variance that is captured by the construct in relation to the amount of variance due to measurement error (Fornell and Larcker, 2016); thus, an AVE value greater than 0.50 indicates that the variance captured by the construct is larger than the amount of variance due to measurement error.

#### Convergent and Concurrent Validity

We found high positive correlations between the CST-HIV dimensions and the validated measures of the same constructs (Table 7). Also, we found correlations in the expected direction between each CST-HIV dimension and the validated instruments used to assess the convergent validity of the other CST-HIV dimensions.

**TABLE 7:** Correlations between the CST-HIV dimensions and the criterion variables.

	Criterion measures											
CST-HIV dimension	SSS	HADS-A	HADS-D	PHQ-4	PROMIS-SEX	DUKE-AC	DUKE-AA	SEI-HS	ISI	FSS	PROMIS-NQ	EQ-5D-5L
STG	0.63**	0.33**	0.19**	0.29**	−0.09	−0.15*	−0.19**	0.21**	0.19*	0.16*	0.12	0.09
EMD	0.35**	0.74**	0.59**	0.71**	−0.32**	−0.32**	−0.36**	0.41**	0.54**	0.42**	0.50**	0.47**
SEX	0.23**	0.34**	0.33**	0.33**	−0.64**	−0.28**	−0.29**	0.35**	0.19**	0.22**	0.20**	0.11
SS	0.23**	0.33**	0.49**	0.28**	−0.38**	−0.59**	−0.64**	0.55**	0.16*	0.11	0.19**	0.27**
MAD	0.22**	0.41**	0.42**	0.37**	−0.30**	−0.36**	−0.42**	0.72**	0.33**	0.19**	0.31**	0.34**
SF	0.19**	0.63**	0.45**	0.52**	−0.30**	−0.25**	−0.28**	−0.32**	0.71**	0.36**	0.41**	0.46**
CG	0.10	0.63**	0.54**	0.58**	−0.26**	−0.33**	−0.32**	0.53**	0.46**	0.46**	0.71**	0.46**
PHYS	0.13	0.59**	0.49**	0.56**	−0.19**	−0.22**	−0.25**	0.31**	0.52**	0.43**	0.52**	0.52**

CST dimensions: STG, anticipated stigma; EMD, emotional distress; SEX, sexuality; SS, social support; MAD, material deprivation; SF, sleep and fatigue; CG, cognitive problems; PHYS, physical symptoms. Criterion variables: SSS, Spanish Stigma Scale; HADS-A, Hospital Anxiety and Depression Scale–Anxiety Subscale; HADS-D, Hospital Anxiety and Depression Scale–Depression Subscale; PHQ-4, Patient Health Questionnaire-4; PROMIS-SEX, PROMIS V2.0 Satisfaction with Sex Life; Duke-AC, Duke-confidential support dimension; Duke-AA, Duke-affective support dimension; SEI-HS, Social Exclusion Index for Health Surveys; ISI, Insomnia Severity Index; FSS, Fatigue Severity Scale; PROMIS-NQ, PROMIS Neuro-QoL V2.0 Cognitive Function; EQ-5D-5L, generic health-related quality of life. Correlations in bold: correlations with specific criterion variables. *p < 0.05, **p < 0.001.

We found negative associations between the eight dimensions of the CST-HIV and the dimensions of HRQoL measured using the disease-specific instrument WHOQOL-HIV-BREF. As can be seen in Table 8, most of the correlations were moderate to high. We also found negative associations between most of the CST-HIV dimensions and the generic measure of HRQoL EQ-5D-5L, with the exception of the anticipated stigma and sexuality dimensions (Table 7).

**TABLE 8:** Correlations between the CST-HIV dimensions and dimensions of health-related quality of life (WHOQOL-HIV-BREF).

	HRQoL dimensions						
CST-HIV dimensions	General health	Physical health	Psychological health	Level of independence	Social relationships	Environmental health	SRPB
Anticipated stigma	−0.18**	−0.25**	−0.27**	−0.17**	−0.27**	−0.28**	−0.52**
Emotional distress	−0.54**	-0.57**	−0.66**	−0.49**	−0.47**	−0.46**	−0.69**
Sexuality	−0.35**	−0.27**	−0.33**	−0.25**	−0.47**	−0.36**	−0.37**
Social support	−0.34**	−0.23**	−0.37**	−0.28**	−0.65**	−0.51**	−0.24**
Material deprivation	−0.46**	−0.33**	−0.35**	−0.42**	−0.44**	−0.68**	−0.27**
Sleep and fatigue	−0.51**	−0.62**	−0.54**	−0.48**	−0.37**	−0.39**	−0.48**
Cognitive problems	−0.47**	−0.58**	−0.64**	−0.57**	−0.42**	−0.44**	−0.49**
Physical symptoms	−0.44**	−0.59**	−0.52**	−0.52**	−0.29**	−0.42**	−0.41**

HRQoL, health-related quality of life; SRPB, spirituality, religion, and personal beliefs. **p < 0.001.

#### CST-HIV Scores

Table 5 reports the CST-HIV dimension scores. These were calculated by adding the values corresponding to each response after recoding the positive items. Thus, higher scores indicate a higher burden in the construct measured in the dimension. All scores were higher than the theoretical mean of the scale except for social support (*M* = 6.95, *SD* = 3.10), although that score was close to it. The highest score was found in the sleep/fatigue dimension (*M* = 9.52, *SD* = 2.97), followed by emotional distress (*M* = 8.90, *SD* = 3.03) and material deprivation (*M* = 8.76, *SD* = 2.80).

## Discussion

The present paper described the development and psychometric properties of a clinic screening tool to facilitate the rapid identification of problems that undermine the HRQoL of PLHIV in Spain. The results indicate that this new measure could be useful for achieving the intended objective. The CST-HIV showed adequate psychometric properties and evidence of content, face, construct and criterion validity. Although this preliminary evidence of validity should be confirmed in a broad validation study, the results enable us to state that a new brief PROM to identify burdensome problems experienced by PLHIV in routine clinical care is now available.

This new instrument has several strengths. It was developed following a robust methodological process that used both qualitative and quantitative data, in accordance with best practices for ensuring content validity (Pedrosa et al., 2014). The selection of the instrument’s content was based on a relevant literature review and on the findings of a qualitative study that included PLHIV and multidisciplinary experts. These procedures allowed us to learn firsthand and from multiple perspectives the problems that undermine the HRQoL of PLHIV in Spain. Findings guided us in determining which issues to prioritise for inclusion in the CST-HIV. The selected issues – anticipated stigma, emotional distress, sexuality, social support, material deprivation, sleep/fatigue, cognitive problems, and physical symptoms – are consistent with research findings about social, psychological, and symptom issues prevalent in Spain (Muñoz-Moreno et al., 2013; Fuster-RuizdeApodaca et al., 2015; Fuster-RuizdeApodaca et al., 2019).

The selected issues are quite similar to those chosen for inclusion in a recent PROM developed by Bristowe et al. (2020) and colleagues on the basis of research conducted in England and Ireland. Those authors have reported the content and face validity of their new instrument. They defined six initial dimensions – physical, cognitive, psychological, welfare, social/relational, and information – and their final version of the instrument is comprised of 23 items. Many of the items are similar to CST-HIV items. However, the other instrument includes some issues that were not considered high priorities by our study participants. These issues included information needs, conception and contraception issues, immigration problems, and alcohol and drug use. Most of these issues also arose during our FGDs, but were not emphasised to the same degree as other issues that we selected for inclusion in our CST-HIV. Several reasons could explain this, such as differences in the epidemiological and socioeconomic profiles of PLHIV whose experiences informed instrument development, differences in the nature of the health-related issues that impose the greatest burden in different settings, and cultural differences that affect how these issues are conceptualised by PLHIV and service providers (Regnault and Herdman, 2015; Nobre et al., 2016). A potential avenue of future research is to explore whether new CST-HIV modules might be developed to add dimensions that are relevant to PLHIV in Spain if this can be done without making the length of the instrument overly burdensome.

After we defined the constructs and drafted the items in accordance with psychometric recommendations, we conducted an inter-judgement process with the participation of 18 multidisciplinary experts, including PLHIV. This process guided us to select and reword items with consideration for clarity, relevance and representativeness. Furthermore, we conducted cognitive debriefing interviews that allowed us to test the face validity of the instrument. According to the previous procedures, the CST-HIV seems to be relevant to, and representative of, the targeted constructs that it is designed to measure, and it is subjectively viewed as covering the concepts that it purports to measure.

The pilot study results enabled us to select a 24-item scale considering both the reliability and validity indices of the items. We were able to estimate the validity indices because our study, despite its pilot nature, included convergent measures for each CST-HIV dimension. We selected three items per dimension, ensuring that both consistency and representation of the construct were fulfilled.

This study also provided preliminary evidence of the validity of the internal structure of the instrument. The results confirmed the eight-factor structure that was theoretically proposed. These factors were related to each other with different magnitudes. The highest covariances were found between the physical symptoms dimension and the dimensions of emotional distress, sleep/fatigue, and cognitive problems. Several studies have found relationships between these issues (Muñoz-Moreno et al., 2014; Tedaldi et al., 2015; Uebelacker et al., 2015; Allavena et al., 2016; Redman et al., 2018; Ren et al., 2018; Nogueira et al., 2019; Sabin et al., 2020). The size of the covariances suggests that these four dimensions could be grouped in a second-order latent dimension that encompasses physical, emotional and cognitive concerns. A second validation study is planned using a larger sample, and in that study it will be feasible to analyse the instrument’s potential second-order structure.

The results showed that most CST-HIV dimensions presented adequate-to-good internal consistency and construct validity. The physical symptoms dimension was the one that showed the lowest internal consistency and construct validity. This result was not surprising because the dimension included three different physical symptoms, with each measured through one item (body changes, pain, and gastrointestinal problems). We decided not to eliminate the dimension for several reasons. The reliability and validity coefficients were not far from the values considered adequate (Bentler, 2009). Moreover, the construct is theoretically relevant. Several studies have shown that the symptoms included in the dimension are prevalent and burdensome (Edelman et al., 2011; Erdbeer et al., 2014; Wilson et al., 2016; Schnall et al., 2018; Ibarra-Barrueta et al., 2019). Additionally, the size of the correlations found between this dimension and other constructs such as HRQoL and psychological well-being endorse its relevance.

This study also provided preliminary evidence of criterion validity of the CST-HIV. We found high correlations between its dimensions and the measures of the convergent constructs. Furthermore, most dimensions presented moderate-to-high correlations with the HRQoL dimensions, providing evidence of concurrent validity. The anticipated stigma dimension was the one that presented the lowest correlations with the criterion measures. The anticipated stigma dimension includes items measuring HIV disclosure concerns and anticipatory fear of being rejected. Previous research on multiple dimensions of stigma has found that the disclosure concerns dimension was less correlated with HRQoL than other dimensions (Franke et al., 2010; Fuster-RuizdeApodaca et al., 2015). A potential explanation for this finding is the mediating role of other variables such as self-efficacy or coping strategies on the negative impact of some stigma dimensions on HRQoL (Fuster-RuizdeApodaca et al., 2015). Although most correlations were small, the anticipated stigma dimension showed a high correlation with the HRQoL domain for spirituality, religion and personal beliefs. This domain of the WHOQOL-HIV-BREF is the one that includes HIV-specific items assessing existential issues and concerns. A previous Spanish study found that the SRPB domain was the unique WHOQOL-HIV-BREF dimension significantly and negatively associated with disclosure concerns (Fuster-RuizdeApodaca et al., 2019). Thus, our current finding provides additional evidence about the relationship between stigma and HIV-specific existential concerns such as those related to fearing the future or feeling that one’s life is meaningful. Correlations found between each CST-HIV dimension and HRQoL point to the relevance of the scale for both theory and intervention.

The present study showed that the scores obtained in most of the CST-HIV dimensions were higher than the theoretical mean of the scale, indicating a relevant burden in these dimensions. The highest scores were found in the sleep/fatigue dimension, followed by emotional distress and material deprivation. These results are consistent with a 2019 Spanish HRQoL study in a cohort of 1462 PLHIV who were demographically similar to the overall Spanish PLHIV population. In that study, sleep was the facet most impaired in the physical health HRQoL dimension, and the psychological HRQoL dimension was one of the most impaired dimensions. The financial resources facet had the lowest score of all facets (Fuster-RuizdeApodaca et al., 2019).

This study had several limitations. We conducted an exploratory but not systematic literature review. Further, our priority in designing the CST-HIV was to keep it brief in order to ensure the feasibility of integrating it into clinical practice. This forced us to prioritise the most prevalent and relevant problems according to our content validity sources. Other potentially relevant health-related issues that negatively impact the HRQoL of PLHIV may have been omitted. It is also possible that the most burdensome problems may change over time in accordance with changing factors such as improvements in ART and simplified ART dosing schedules. To offset these limitations, we recommend collecting HRQoL data periodically to assess whether other dimensions will emerge as more burdensome. The desired brevity of the measure led us to choose only three items in each dimension. This could result in low levels of reliability and low construct validity scores in some dimensions. It might also have an impact on the predictive validity of the tool. We plan to test it further in subsequent studies, and we anticipate that by defining risk cut-off points for the scores on all dimensions, we will be able to provide guidance to healthcare providers regarding when findings should be followed up with the administration of other validated PROMs to further investigate specific issues of concern. Moreover, PLHIV are a heterogeneous group, and there are specific sub-groups particularly vulnerable to poor HRQoL (Degroote et al., 2014; Fuster-RuizdeApodaca et al., 2019). Thus, we should analyse the scale invariance as a function of relevant sociodemographic or epidemiological characteristics. This would allow for the generalisation of the model (Vandenberg and Lance, 2000). Moreover, this scale was developed and tested in the Spanish context. Thus, the scale and its factor structure should be tested in samples from other cultures to investigate its applicability in different contexts. As a first step, we will perform the cross-cultural adaptation of the CST-HIV to another European country (Italy).

Despite these limitations, we can conclude that we have a new brief instrument to screen eight significant problems that undermine HRQoL and contribute to poor health outcomes in PLHIV. The CST-HIV appears to have good psychometric properties and good preliminary evidence of validity. We anticipate that our next validity study results will strengthen the present evidence, recommending its use in clinical care in Spain. In addition to conducting the CST-HIV validation study, our other planned research will involve assessing the usefulness, efficacy, feasibility, and acceptability of integrating the CST-HIV and related PROMs into clinical practice.

The use of PROMs has been associated with improvements in clinical care and in health outcomes in fields such as mental health and oncology, and there are unrealised opportunities for the HIV field to integrate PROMs into clinical care in ways that will benefit patients (Fredericksen et al., 2020b; Kall et al., 2020). This new instrument is particularly timely in light of growing interest in the objective of improving HRQoL in PLHIV (Lazarus et al., 2016; Guaraldi et al., 2019). Our research findings are novel because few studies focus on brief screening PROMs that cover the range of biological, psychological and social issues that impair the HRQoL of PLHIV, and the present study is unique in Spain. The clinical care challenges presented by the COVID-19 pandemic underscore the importance of implementing tools that will help PLHIV and their healthcare providers make the best use of limited consultation time (Guaraldi et al., 2020). Using the CST-HIV to gather information about patients’ symptoms, concerns, and experiences in advance of clinical appointments could help determine individual consultation models, resulting in greater patient satisfaction and better health outcomes.

## Data Availability Statement

The datasets presented in this study can be found in online repositories. The names of the repository/repositories and accession number(s) can be found below: Figshare repository [https://doi.org/10.6084/m9.figshare.14216162].

## Ethics Statement

The studies involving human participants were reviewed and approved by the Ethics Committee of the Hospital Clínic of Barcelona. The patients/participants provided their written informed consent to participate in this study.

## Author Contributions

KS-H and MJFR conceptualised the study, prepared the focus group scripts, and prepared the first draft of the manuscript. KS-H, MJFR, and JL jointly acquired funding for the study and organised the focus group discussions. MJFR formulated the study design. KS-H conducted literature reviews. MJFR moderated the focus groups with assistance from JL. MJFR conducted the pilot study and was the main analyst with the support of AL. MJFR performed the qualitative analysis of focus group data and reported on results. KS-H, MJFR, and MP contributed to the interpretation of results and participated in the development of the pool of items. MJFR, MP, and DN were involved in the inter-judgement process. All authors participated in revising the manuscript and all authors approved the final manuscript.

## Conflict of Interest

MJFR and MP are members of the Spanish Interdisciplinary AIDS Society (SEISIDA) Executive Board. SEISIDA has received grants from Gilead, Janssen, MSD and ViiV, outside of the submitted work. MJFR has provided consultancy services to Gilead, Janssen, MSD and ViiV, and has received payments for lectures or educational presentations from Gilead, Janssen, MSD and ViiV, outside of the submitted work. JL reports grants and speaker fees from AbbVie and MSD, outside of the submitted work, speaker fees from Gilead Sciences and ViiV, and an institutional grant from Gilead Sciences for this study. The remaining authors declare that the research was conducted in the absence of any commercial or financial relationships that could be construed as a potential conflict of interest.

## Footnotes

1 www.qualtrics.com

## Supplementary Material

The Spanish Clinic Screening Tool (CST-HIV) items are presented in both Spanish and English in Supplementary Table 1.
