# Supplementary material for: Development of a Clinic Screening Tool to Identify Burdensome Health-Related Issues Affecting People Living With HIV in Spain
Source: Front Psychol. 2021 Jun 9;12:681058. doi: 10.3389/fpsyg.2021.681058 (PMC8219862; doi:10.3389/fpsyg.2021.681058)
Supplement: Supplementary file 1 [file Table_1.docx]

**Supplementary Table 1.** Spanish and English versions of the CST-HIV items

| Nº | Item code number | Item in Spanish | Item in English | Dimension |
| --- | --- | --- | --- | --- |
| 1 | S1 | En el último mes, ¿hasta qué punto te ha preocupado contarle a alguien que tienes VIH? | During the past month, to what extent have you been worried about telling someone you have HIV? | Anticipated stigma |
| 2 | S3 | En el último mes, ¿hasta qué punto te ha preocupado que la gente te juzgue si se enteran que tienes VIH? | During the past month, to what extent have you been worried about people judging you if they learn you have HIV? |  |
| 3 | S5m | En el último mes, ¿hasta qué punto te ha preocupado pensar que la gente te va a rechazar por tener VIH? | During the past month, to what extent have you been worried about people rejecting you for having HIV? |  |
| 4 | E2m | En el último mes, ¿con que frecuencia has sentido ansiedad? | During the past month, how often have you felt anxiety? | Emotional distress |
| 5 | E3m | En el último mes, ¿con que frecuencia has tenido sentimientos de tristeza o decaimiento? | During the past month, how often have you felt sadness or felt discouraged? |  |
| 6 | E5 | En el último mes, ¿con que frecuencia has sentido temor al futuro? | During the past month, how often have you felt fearful of the future? |  |
| 7 | Sx1m | En el último mes, ¿en qué medida te has sentido satisfecho/a con tu vida sexual?^1^ | During the past month, how satisfied have you felt with your sex life?^1^ | Sexuality |
| 8 | Sx2 | En el último mes, ¿en qué medida ha disminuido tu deseo sexual o tu interés en tener sexo? | During the past month, has your sex drive or your interest in sex decreased? |  |
| 9 | Sx6 | En el último mes, ¿en qué medida ha afectado el VIH negativamente a tu vida sexual? | During the past month, has HIV negatively affected your sex life? |  |
| 10 | SS1m | En el último mes, ¿con que frecuencia has tenido personas a tu alrededor en las que apoyarte en caso de necesitarlo?^1^ | During the past month, how often have you had people around you whom you can lean on in case of need?^1^ | Social support |
| 11 | SS2 | En el último mes, ¿con que frecuencia has tenido alguien de confianza para hablar de tus problemas?^1^ | During the past month, how often have you had someone you trust to speak to about your problems?^1^ |  |
| 12 | SS3 | En el último mes, ¿con que frecuencia has tenido gente que te haya hecho sentirte querido/a?^1^ | During the past month, how often have people made you feel loved?^1^ |  |
| 13 | Ex3 | En el último mes, ¿hasta qué punto has tenido suficiente dinero para cubrir tus necesidades?^1^ | During the past month, have you had enough money to meet your needs?^1^ | Material deprivation |
| 14 | Ex4m | En el último mes, ¿hasta qué punto has estado satisfecho/a con la calidad del lugar donde vives?^1^ | During the past month, how satisfied have you been with the quality of the place where you live?^1^ |  |
| 15 | Ex5m | En el último mes, ¿hasta qué punto has tenido dinero para realizar actividades de ocio?^1^ | During the past month, have you had money for leisure activities?^1^ |  |
| 16 | SF1 | En el último mes, ¿hasta qué punto has tenido problemas de sueño? | During the past month, have you had sleep problems? | Sleep and fatigue |
| 17 | SF7 | En el último mes, ¿hasta qué punto te has sentido satisfecho/a con la calidad de tu sueño?^1^ | During the past month, how satisfied have you felt with the quality of your sleep?^1^ |  |
| 18 | SF8 | En el último mes, ¿hasta qué punto te has sentido cansado/a? | During the past month, how tired have you felt? |  |
| 19 | CG4 | En el último mes, ¿hasta qué punto has tenido dificultad para pensar con claridad? | During the past month, have you had difficulty thinking clearly? | Cognitive problems |
| 20 | CG7 | En el último mes, ¿hasta qué punto has tenido dificultad para prestar atención? | During the past month, have you had difficulty paying attention? |  |
| 21 | CG8 | En el último mes, ¿hasta qué punto crees que te ha costado más aprender cosas nuevas? | During the past month, do you think that it has been harder for you to learn new things? |  |
| 22 | PS1m | En el último mes, ¿hasta qué punto has tenido cambios molestos en tu apariencia corporal, como acúmulos de grasa, ganancia o pérdida de peso? | During the past month, have you experienced unpleasant body changes such as fat accumulation, weight gain, or weight loss? | Physical symptoms |
| 23 | PS3 | En el último mes, ¿hasta qué punto has tenido dolor en alguna parte del cuerpo? (por ejemplo, dolor de cabeza, articulaciones, pinchazos en los músculos, etc.) | During the past month, have you felt pain somewhere in your body? (for example, headache, joint pain, muscle cramps) |  |
| 24 | PS5 | En el último mes, ¿hasta qué punto has tenido molestias digestivas (dolor de estómago, gases, diarrea, nauseas o vómitos) | During the past month, have you suffered digestive problems? (stomach pain, flatulence, diarrhea, nausea, or vomiting) |  |

^1^ Reverse item
